# Comprehensive Cancer Control in the Eye of Hurricane Katrina

**Published:** 2009-09-15

**Authors:** Donna Williams, Randi Kaufman, Jennifer Hayden, Melody Robinson, Eric Tai

**Affiliations:** Louisiana State University Health Sciences Center, New Orleans, Louisiana; Louisiana State University Health Sciences Center, New Orleans, Louisiana; Louisiana State University Health Sciences Center, New Orleans, Louisiana; Louisiana State University Health Sciences Center, New Orleans, Louisiana; Centers for Disease Control and Prevention, Atlanta, Georgia

## To the Editor:

In August 2005, Hurricane Katrina caused widespread damage to New Orleans and the surrounding area (1). Despite this damage, Louisiana's Comprehensive Cancer Control Program (CCCP) remained functional because of a decentralized management structure and strong partnerships. Lessons learned from this experience can be applied to basic emergency response planning.

The Louisiana CCCP operates from the Louisiana State University Health Sciences Center School of Public Health. The CCCP, in cooperation with its primary partners, the Louisiana chapter of the American Cancer Society, the Campaign for a Tobacco Free Louisiana, and the Louisiana Office of Public Health, created the Louisiana Cancer Control Partnership (LCCP) in 2003 to oversee development and implementation of the Louisiana CCCP and reduce the burden of cancer in the state. LCCP comprises more than 200 organizations and implements comprehensive cancer control activities in Louisiana (2). LCCP's structure consists of a management team, an executive committee, and regional coalitions. LCCP has a decentralized management structure in which decision making occurs centrally and regionally whereas implementation occurs locally. The management team is based in New Orleans and guides overall development of comprehensive cancer control in the state. The executive committee of state experts provides input on the development of statewide goals and objectives. Louisiana has 9 regional coalitions based on 9 health regions. These regional coalitions formulate and implement local action plans on the basis of statewide goals and objectives.

New Orleans was declared uninhabitable due to flooding on August 31, 2005, 2 days after Hurricane Katrina made landfall in Louisiana (3). LCCP's New Orleans offices were flooded, and staff evacuated the city. In the first months after Katrina, only one-third of the staff was able to return. In the New Orleans area, communication channels were nonfunctional (3).

Strong prestorm partnerships allowed comprehensive cancer control activities to continue in Louisiana after Hurricane Katrina. In October 2005, several LCCP staff relocated to a partner organization's offices in the Baton Rouge area. Partnerships also allowed professional education on cancer control to continue in the state with minimal interruption. Before Hurricane Katrina, professional education had been broadcast by LCCP staff from New Orleans. Because storm damage made broadcasts impossible, a regional partner in Shreveport assumed this function. Decentralization of comprehensive cancer control activities in the state allowed the management team to focus on Hurricane Katrina–related medical and public health issues in New Orleans. Comprehensive cancer control in the state was not dependent on the management team for day-to-day direction because local action plans were designed and implemented regionally. Hurricane Katrina directly affected only 1 of the 9 regions in Louisiana. Therefore, regional coalitions continued local comprehensive cancer control activities in the 8 regions of the state unaffected by Hurricane Katrina. This continuation resulted in minimal disruption to comprehensive cancer control activities in most of the state.

Decentralized management and strong partnerships through LCCP were essential to effective operation of comprehensive cancer control activities during and after Hurricane Katrina. In response to the devastation caused by Hurricane Katrina, CCCP has developed emergency communication and logistics plans to allow staff to communicate and work in the event of a disaster (Table). Staff members now have multiple ways to communicate, including e-mail, text messaging, use of conference lines, and through Web sites. Staff have been issued laptop computers and portable document storage to allow functionality during an emergency. A Google site with a calendar and uploaded documents is accessible to staff who have Internet access, and it functions as a virtual meeting place for staff after a disaster.

Hurricanes affecting Louisiana in September 2008 were the first opportunities to assess whether these emergency plans would function as envisioned. Although the management team in New Orleans carried out the plans and continued functioning, some regional coalitions outside of New Orleans did not. Comprehensive cancer control activities ceased in 2 regions of the state for several weeks when offices flooded and basic utilities were unavailable. All regions in the state will be asked to consider these experiences and implement emergency communication and logistics plans.

Despite the damage from Hurricane Katrina, comprehensive cancer control activities continued in Louisiana. A decentralized structure and strong partnerships allowed state comprehensive cancer control to continue through regional coalitions in areas not directly affected by Hurricane Katrina. Lessons learned from these experiences have shaped emergency plans that enable continued functioning of comprehensive cancer control programs during disasters in coordination with statewide partners.

## Figures and Tables

**Table.** Suggestions to Improve Emergency Preparedness of Comprehensive Cancer Control Programs


**Structure of Comprehensive Cancer Control Program**

• Decentralize comprehensive cancer control planning and functions to allow work to continue in areas not affected by disaster.
• Build strong partnerships inside and outside your local area through communication and collaboration on comprehensive cancer control activities. Arrange reciprocal relationships to have data or functions transferred in case of emergencies.

**Emergency Communication and Logistics**

• Build redundancy into communication plans (eg, identify multiple contacts for staff and partners, cross train staff).
• Use Internet-based communication, such as Web sites, e-mail, and online spaces during and after a disaster. Direct staff to share information and updates by using these tools.
• Issue adequate equipment to staff, including laptop computers and portable document storage, to use in the event of evacuation.
• Ensure that disaster planning includes the time before and after the disaster. Specify time frames and identify people responsible for all activities.
• Review and update plans at least annually.
• Pilot test plans, if possible.
